# Glances at the Hospitals

**Published:** 1904-10-22

**Authors:** 


### GLANCES AT THE HOSPITALS.

### GREENOCK HOSPITAL AND INFIRMARY.

The ordinary income was £6,368, and the ordinary expenditure was £6,367. The extraordinary income was £1,381, and the extraordinary expenditure was £99, showing a surplus of £1,282. The committee were able to carry £1,083 to the reserve fund and £200 to their funded account. The ordinary subscriptions were £830, and the workpeople contributed £849. We failed to notice any statement of the cost per bed occupied or the cost per patient. On January 1st the hospital contained 102 patients, and 1,009 were admitted during the year, making a total of 1,111 under treatment in this department. Of this number 1,006 were treated to a termination with the following results: recovered 703, relieved 128, discharged for other reasons 66, and died 109. In the fever house 74 remained at the beginning of the year, and 306 were admitted. Of the total number, 318 were discharged recovered, 9 relieved, and 28 died. Of 185 cases of scarlet fever 150 were cured, 4 died, and 31 remained in the wards, and of 89 cases of enteric fever, 69 were cured, 18 died, and 2 remained. From the statistical tables of the hospital we extract the following: Of 42 cases of pneumonia 23 were cured and 19 died. Of 6 cases of strangulated hernia, 3 were cured, 2 died, and 1 remained. A table of the deaths is given which shows the disease causing death, the sex and age of the patient, and the duration of treatment in the hospital. It may be said that the tables are all very clearly arranged, but we have to note the absence of an anæsthetic table.

### THE OLDHAM INFIRMARY.

The annual income was £5,601, and the annual expenditure was £5,561, showing a credit balance of £40. This satisfactory result has been obtained by the generosity of many of the subscribers who have increased their contributions, and by the Hospital Saturday Fund collected weekly in the mills and workshops which amounted to £1,640. Extensive additions and alterations have been carried out, and the hospital is now described as being "as large as the present site will allow, and up to date in all particulars." Hitherto an income of less than £6,000 has sufficed to keep the institution going, but when the extensions are in use the income required will be nearly £7,000, and the committee close their report with the expression of a hope that the people of Oldham will not allow the increased usefulness of the hospital to be crippled for lack of funds. The cost per bed occupied is not stated, nor the cost per patient. There were 660 patients admitted during the year and 57 remained under treatment at the end of it. Of the total number 432 were discharged recovered, 119 were discharged relieved, 38 for other reasons, and 69 died; 356 operations were performed in the theatres, and 200 minor operations were performed in the wards or accident-room; but no anæsthetic table is given so we are in ignorance of the agents used. There were 13 cases of appendicitis, of whom 9 were discharged, 2 died, and 2 remained in the wards. Cæsarian section was performed 3 times, all being successful. Among the injuries we note 8 cases of fracture of the base of the skull, 6 being fatal and 2 recovered.

### VICTORIA HOSPITAL, FOLKESTONE.

The total income for the year was £2 930, and the expenditure was £2,906. The committee were therefore able to reduce the adverse balance which remained from the previous year. The average cost per bed occupied was £82 6s. 6d., the average cost per patient was £5 19s. 6d., and the average cost of provisions per head, including staff, was 6s. 4½d. a week. At the beginning of the year 34 patients were in residence, and 413 were admitted during the year, making a total of 447 under treatment. Of these, 360 were discharged cured or relieved, 21 as incurable, 32 died, and 34 remained in the wards. There were 116 major operations and 131 minor operations performed during the year. Among the major operations were 28 cases of abdominal section, and it would have been interesting and instructive to learn something about the conditions requiring operation and the results, but no medical or surgical tables are given, an omission which we hope to see remedied in next report. There is a table showing the causes of death in the

32 patients dying during the year, and a statement that the death-rate was 7·1 per cent.

THE BLACKBURN INFIRMARY.

The total income was £7,408, and the total expenditure was £7,263. The endowment fund has been increased by a sum of £3,000 from the executors of the late John Pickop and by a donation of £1,000 from Mrs. Fielden. Collections at the mills and workshops amounted to £2 393, fees for private nursing to £408, and probationers' fees to £63. A theatrical entertainment produced £126, and the infirmary ball £62. Including 89 patients in residence at the beginning of the year the number treated was 1,602. The average duration of treatment was 24 days. The total number of deaths was 93, giving a death-rate of 6·85 per cent., but if 37 occurring within 48 hours of admission be deducted, the percentage falls to 4 4. There were 35 cases of acute and sub-acute rheumatism, and all of these recovered. Of pneumonia and broncho-pneumonia there were 13 cases, of whom 8 recovered and 5 died. Of 13 operations for strangulated hernia 8 recovered and 5 died. Of 38 amputations all were successful. Of 2 cases of ovariotomy both were cured. The total number of operations was 560, and there were 27 deaths. No anæsthetic table is given.

